# Quality and reliability of knee osteoarthritis-related information on short video platforms in China: a multi-method cross-sectional study

**DOI:** 10.1186/s12889-026-26455-9

**Published:** 2026-02-02

**Authors:** Xinwu Duan, Yan Wang, Tiancheng Ma, Mengfan Cai, Jianxiong Ma, Xinlong Ma

**Affiliations:** 1 Tianjin Hospital Of Tianjin University (Tianjin Hospital), Tianjin, 300211 China; 2 Tianjin University of Sport, Tianjin, 301617 China; 3 Tianjin Orthopedic Institute, Tianjin, 300050 China; 4 Tianjin Key Laboratory of Orthopedic Biomechanics and Medical Engineering, Tianjin, 300050 China

**Keywords:** Knee osteoarthritis, Short videos, Reliability, Content completeness, Health information quality

## Abstract

**Background:**

Knee osteoarthritis is a highly disabling chronic disease that imposes a substantial societal burden.Short video platforms have become a primary source of health information in China, yet the quality of such content is highly variable. There is currently a lack of systematic and multi-platform assessments of knee osteoarthritis-related information quality on these platforms.

**Method:**

This study retrieved and included 300 videos related to knee osteoarthritis from three platforms—TikTok, Bilibili, and Rednote—as the analytical sample. The basic characteristics of the videos (likes, comments, duration, etc.), the identities of the publishers (orthopedic surgeons, other medical personnel, institutions, and ordinary users), and the content types were collected and analyzed with descriptive statistics. Three standardized tools, Global Quality Scale (GQS), modified DISCERN (mDISCERN), and The Journal of the American Medical Association (JAMA), were used to independently assess the quality of the videos. We also examined the correlation between these quality scores and video features.

**Result:**

There were significant differences among the three platforms. TikTok videos had the highest user engagement but the shortest duration (median 108 s); Bilibili had the longest video duration (median 262 s), and achieved a significantly higher median GQS score 3(IQR2-4) than Rednote 2(IQR2-3) (*p* < 0.001); Rednote performed the best and was the most stable in JAMA scores (*p <* 0.01). The quality scores of videos released by medical professionals and institutions were significantly higher than those of ordinary users in all assessment tools (*p* < 0.01). Correlation analysis showed that video quality (GQS, mDISCERN, JAMA) was only weakly or insignificantly correlated with the popularity indicators (likes, followers, etc.), but a positive correlation trend with video duration.

**Conclusion:**

The generally suboptimal quality of knee osteoarthritis information on Chinese short-video platforms and its disconnect from popularity metrics highlight a growing public health concern. As short videos increasingly serve as a key source of health information, it is imperative to strengthen content quality oversight. Collaborative efforts among platforms, health authorities, and the public are essential to improve the reliability of online health content and support public access to accurate health information.

**Supplementary Information:**

The online version contains supplementary material available at 10.1186/s12889-026-26455-9.

## Introduction

 Knee osteoarthritis (KOA) is a chronic disease characterized mainly by the degeneration of joint cartilage and bone hyperplasia. It typically manifests as pain, stiffness, and functional impairment [1]. As a leading global cause of disability, KOA affects an estimated 300 million individuals, most commonly in the knee joint [2, 3]. The disease severely compromises patients’ quality of life and imposes a substantial socioeconomic burden [4]. Effective management and early intervention depend critically on public understanding of KOA. However, raising disease awareness and ensuring access to trustworthy information pose persistent challenges.

With the widespread adoption of digital technology, short videos have become the primary means for the public to obtain information. In China, short-video platforms serve over 1.05 billion users [5]. This shift extends to healthcare, with approximately 80% of individuals seeking health information online [6], and short videos playing an increasingly crucial role in health education [7]. However, the quality of such content is highly variable, and issues like information asymmetry and poor reliability are widespread [8–12].This situation presents a critical public health dilemma: patients managing chronic conditions like KOA are increasingly dependent on digital sources for guidance, yet the quality of this information is largely unregulated and uncertain.

Mainstream short-video platforms in China—TikTok (Douyin), Bilibili, and Rednote (also known as Xiaohongshu)—reach users across all age groups [13–15]. This widespread reach is particularly significant in a country where approximately 18% of the population suffers from KOA [16]. As of May 2025, these platforms commanded substantial monthly active user bases: approximately 1.022 billion for TikTok [17], 368 million for Bilibili [14] and 242 million for Rednote [15]. While previous studies have assessed video quality for conditions like cancer and spinal disorders [18–22], a systematic, multi-platform analysis of KOA-related content on mainstream Chinese platforms is lacking. This gap highlights a critical need for evidence to guide the public in evaluating the reliability of the vast amount of KOA information they encounter online.

To address this gap, we conducted a systematic and multi-platform comparative analysis of Chinese short videos related to KOA. Using standardized assessment tools, including the Global Quality Scale (GQS), we evaluated the quality and reliability of KOA-related short videos on TikTok, Bilibili, and Rednote.Specifically, this study aimed to achieve the following objectives: (1) Systematically evaluate and compare the quality of KOA information across multiple mainstream Chinese platforms; (2) Examine the relationship between information quality and popularity metrics, and assess differences across content publisher types; (3) Synthesize evidence-based insights that can inform better practices for content creation and public health communication. Through this multi-platform comparison, our research seeks to empower users to critically evaluate KOA content and to provide concrete evidence for strategies aimed at enhancing the reliability of online health information.

## Materials and methods

### Search strategy and data collection

**Fig. 1:**
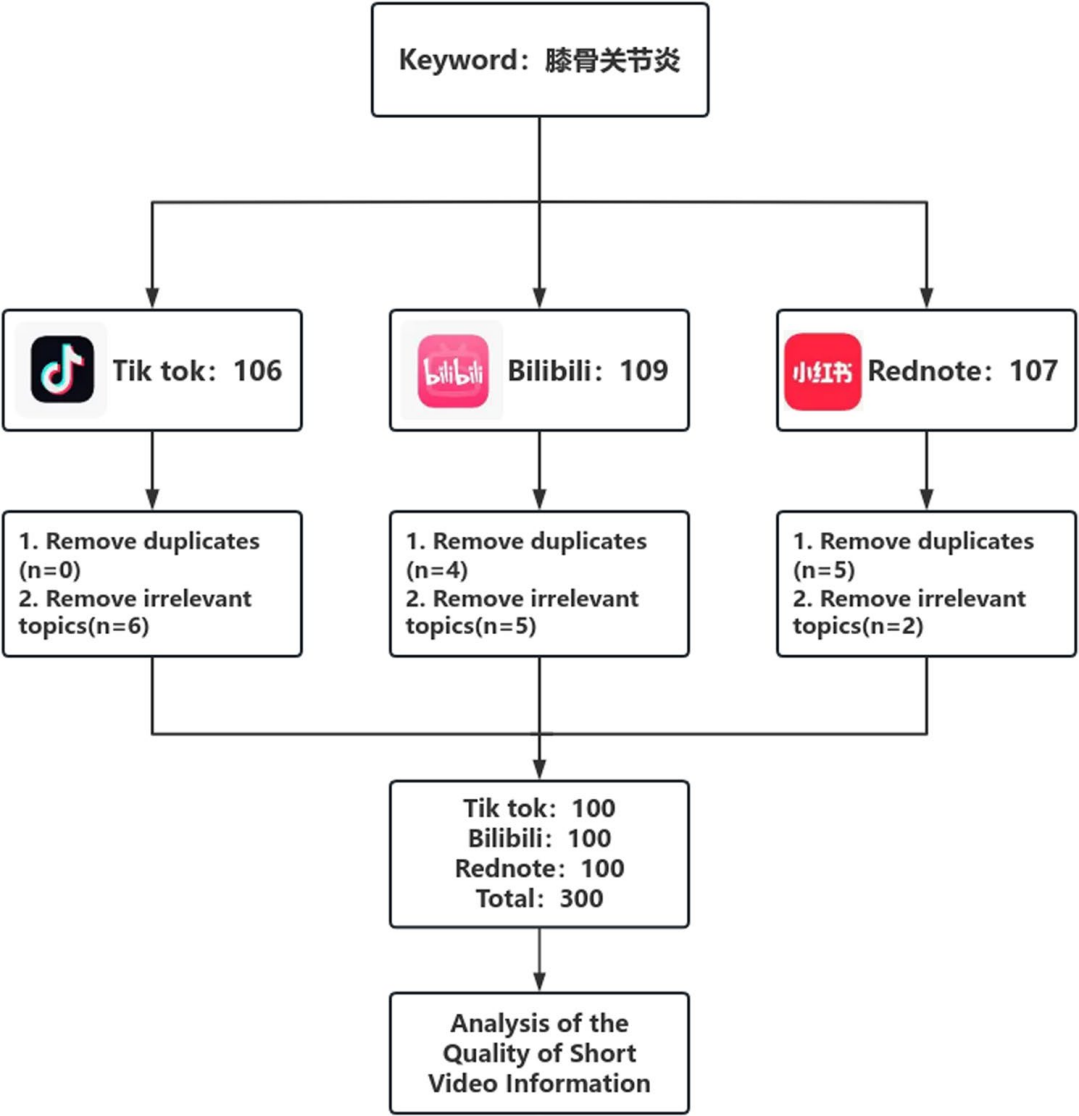
Short-video retrieval strategy and screening process

Data were collected on September 1, 2025. To minimize bias from personalized recommendation algorithms, we registered new, neutral user accounts (with no prior browsing history) on three platforms: TikTok (https://www.douyin.com/), Bilibili (https://www.bilibili.com/), and Rednote (https://www.xiaohongshu.com/). A researcher uniformly used the term “膝骨关节炎” (Chinese: knee osteoarthritis) as the search keyword to search content across the three short-video platforms.Videos were screened in the default sorting order, as this reflects the primary browsing behavior of users and ensures that the sample represents the content most commonly encountered in real-world use.The exclusion criteria included: (1) content completely unrelated to knee osteoarthritis; (2) duplicate videos within the same platform, in which case only the first occurrence in the sorting order was retained. Following the initial order of the default sorting, the top 100 eligible short videos from each platform were selected. Following the exclusion of duplicate entries and irrelevant content, a total of 300 videos were finalized as the analytical sample. The detailed screening process is illustrated in Fig. 1.

### Classification of short video information

We collected the following metadata for each of the 300 videos: the number of likes, comments, favorites, shares, and followers, as well as video duration. Based on core thematic content, each video was categorized into one of six types: disease education and assessment, surgical treatment, drug therapy, anatomical pathology, patient case sharing, and news advertisements (For specific classification criteria, please refer to Additional file 4). Uploaders were classified into four groups based on account attributes: orthopedic surgeon (orthopedic physicians with professional qualification certifications), other medical personnel (e.g. physical therapists, nurses.), institutions (hospitals, KOA education and publicity institutions), and ordinary users (personal accounts without relevant professional certification).

### Examination tools and evaluation

In this study, Global Quality Scale (GQS), modified DISCERN (mDISCERN), and The Journal of the American Medical Association (JAMA), were used to evaluate the quality and reliability of KOA videos on three short video platforms (see Supplementary Materials for details). The core dimensions of these standardized tools—information quality, reliability, and transparency—demonstrate cross-media applicability and have been adapted and validated within the context of health-related short video research [18, 20, 23]. Moreover, in the context of Chinese short videos, previous studies have verified and utilized these three standardized tools to conduct evaluations of the information quality and reliability of health-related short videos [24–26].

GQS provides a global assessment of the overall quality and utility of health education videos using a 5-point ordinal scale (1 = very poor quality, 5 = excellent quality) [23].

mDISCERN is a simplified instrument derived from the DISCERN tool, which is designed to assess the quality of health information, particularly regarding treatment choices [27]. The mDISCERN version focuses on the reliability and objectivity of information, evaluating five core aspects: clarity of aims, objectivity of evidence, reliability of sources, provision of additional information, and acknowledgment of uncertainty. Each item is scored dichotomously (1 = meets criterion, 0 = does not meet criterion) [28].

JAMA assesses the fundamental credibility of health information based on four standards related to transparency and timeliness: authorship, attribution, currency, and disclosure of conflicts of interest [29]. Each item is scored 1 point if it meets the criteria and 0 points if it does not.

Three inspectors conducted the inspection and evaluation of the video. They were all professionals who had been engaged in the research of orthopedic diseases for a long time. Prior to the evaluation, all three inspectors received training on the GQS, mDISCERN, and JAMA tools. Among them, two evaluators assessed the content quality and reliability of the videos across the three platforms. Weighted Kappa coefficients and ICC were used to confirm good consistency between the two evaluators’ ratings. Inter-rater reliability was excellent: for GQS scores, the weighted Kappa was 0.832 (95% CI: 0.78–0.88); for mDISCERN and JAMA scores, the intraclass correlation coefficients (ICC) were 0.802 (95% CI: 0.75–0.85) and 0.922 (95% CI: 0.90–0.94), respectively(see Additional file 4 for specific details). In cases of disagreement between the two evaluators, the third evaluator participated in the discussion to reach a final consensus.

### Statistical analysis

Statistical analyses were performed using GraphPad Prism 10.1 and IBM SPSS 27.0. Descriptive statistics were first conducted on the collected short videos. Video metadata (e.g., number of likes, comments, collections, duration) were presented as median with interquartile range (IQR). Mean values with standard deviations were also reported for the GQS, mDISCERN, and JAMA scores. The normality of the data was assessed using the Shapiro‑Wilk test. As all data were non‑normally distributed, comparisons among three or more independent groups were performed using the Kruskal‑Wallis test. If the Kruskal-Wallis test was significant, Dunn’s post-hoc test with adjusted p-values was applied for pairwise comparisons. Correlations between variables were evaluated using Spearman’s rank correlation analysis. All statistical analyses were considered statistically significant at *p* < 0.05.

## Results

### Statistics of basic information and quality scores of short videos

We conducted descriptive statistics on 300 KOA-related short videos from three platforms, as presented in Table 1. The median (IQR) for likes, comments, favorites, shares, followers, and video duration were 236 (36–2278), 20 (5–102), 193 (29–1278), 86 (14–627), 22,000 (4271–182000), and 131 (72–262), respectively.Comparative analysis across the platforms showed that TikTok had the highest number of likes, comments, favorites, shares, and followers for KOA-related short videos, while Bilibili had the longest video duration. These results suggest that KOA-related short video content reaches a wider audience and generates higher engagement on TikTok.Analysis of the collected scoring data revealed that, as presented in Tables 1 and 2, the overall quality scores of videos across the three platforms were not high, and uploader identity had a significant impact on video quality scores.

**Table 1:** Basic characteristics and quality scores of the videos

Variables	Total (*n* = 300)	TikTok (*n* = 100)	Bilibili (*n* = 100)	Rednoten (*n* = 100)	*P*
Basic Information					
Likes, M(IQR)	236,(36-2278)	3860,(717-13750)^a^	64,(15–266)	122,(27–386)	< 0.001
Comments, M(IQR)	20,(5-102)	171,(37–695)^a^	8,(3–24)	10,(4–32)	< 0.001
Collections, M(IQR)	193,(29-1278)	2127,(251–7418)^a^	127,(22–423)	68,(20–299)	< 0.001
Shares, M(IQR)	86,(14–627)	1161,(96-5090)^a^	37,(6-136)	30,(11–199)	< 0.001
Fans, M(IQR)	22,000,(4271-182000)	221,000,(57750–805000)^a^	7709,(1210–52500)	15,000,(5830–29000)	< 0.001
Durations, M(IQR)	131,(76–262)	108,(65–208)	262,(145–454)^c^	92,(57–149)^b^	< 0.001
Short video quality scores					
GQS, M(IQR)	3,(2–3)	3,(2–3)	3,(2–4)	2,(2–3)	< 0.001
GQS, mean ± SD	2.75 ± 0.95	2.74 ± 1.08	2.99 ± 0.89	2.52 ± 0.80	
mDISCERN, M(IQR)	3,(2–3)	2,(2–3)	3,(2–3)	2,(2–3)	0.131
mDISCERN, mean ± SD	2.53 ± 0.79	2.44 ± 0.97	2.63 ± 0.69	2.52 ± 0.67	
JAMA, M(IQR)	2,(1–2)	2,(1–2)	2,(1–2)	2,(2–2)	< 0.011
JAMA, mean ± SD	1.93 ± 0.69	1.81 ± 0.84	1.94 ± 0.72	2.05 ± 0.44	

*M* Median, *IQR* The interquartile range, Inter-group comparisons of short video data across the three platforms were performed using the Kruskal–Wallis test, followed by Dunn’s test for multiple comparisons.

^a^ indicates a score significantly higher than those of the other two platforms

^b^ indicates a score significantly higher than that of one platform

^c^ indicates no significant difference from the other two platforms

**Table 2:** Scores by uploader identity

Variables	Orthopaedic Surgeon	Other Medical personnel	Ordinary user	Institution	*P*
GQS, M(IQR)	3,(2–3)	3,(3–4)	2,(2–3)	3,(2–4)	< 0.001
GQS, mean ± SD	2.75 ± 0.93	3.13 ± 0.78	2.17 ± 0.83	3.00 ± 1.00	
mDISCERN, M(IQR)	3,(2–3)	3,(2–3)	2,(1.5-3)	2,(2–3)	< 0.001
mDISCERN, mean ± SD	2.54 ± 0.73	2.87 ± 0.73	2.01 ± 0.80	2.71 ± 0.79	
JAMA, M(IQR)	2,(2–2)	2,(2–3)	1,(1–2)	2,(1.25-2)	< 0.001
JAMA, mean ± SD	2.00 ± 0.62	2.22 ± 0.69	1.40 ± 0.57	2.00 ± 0.75	

Comparisons of scores across different uploader identities were performed using the Kruskal–Wallis test

### The proportion of publisher identities and types of published content

**Fig. 2:**
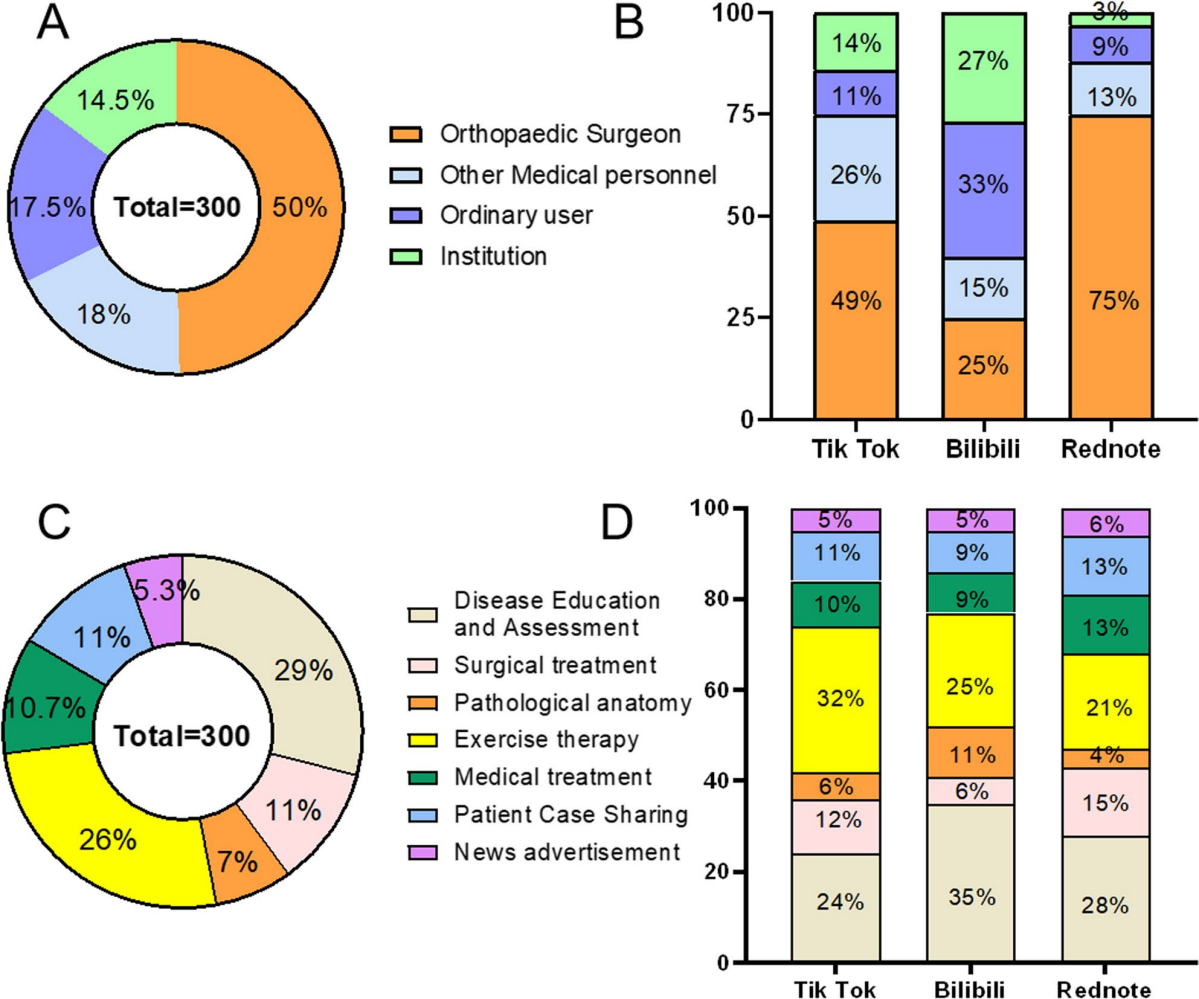
**A** the total proportion of different publisher identities on the short video platform; **B** the proportion of different publisher identities on TikTok/Bilibili/Rednote platform; **C** the proportion of total content released by short video platforms: **D** the proportion of different content released by TikTok /Bilibili/Rednote platforms

Statistical analysis of the 300 videos showed that orthopedic surgeons and other healthcare professionals accounted for approximately 50% (149/300) and 18% (54/300) of uploaders, respectively, while general users and institutions represented 17.5% (53/300) and 14.5% (44/300). Overall, medical professionals contributed 68% (193/300) of the videos, exceeding the share posted by non-medical users (Fig. 2A).

As shown in Fig. 2B, medical professionals (orthopedic surgeons and other healthcare personnel) accounted for the majority of uploaders on TikTok (75%, 75/100) and Rednote (88%, 88/100). In contrast, on Bilibili, only 40% (40/100) of videos were published by medical professionals, indicating a higher proportion of non-medical contributors on this platform.

Regarding content categories (Fig. 2C), disease education and assessment (29%, 87/300) and exercise therapy (26%, 78/300) were the most common topics, followed by surgical treatment, pharmacological treatment, and patient case sharing. Pathological anatomy and news/advertisements were less frequently covered.

A platform-specific comparison (Fig. 2D) revealed that exercise therapy videos were most prevalent on TikTok (32%, 32/100). On Bilibili, disease education/assessment (35%, 35/100) and pathological anatomy (11%, 11/100) were the leading categories. Rednote had the lowest share of pathological anatomy content (4%, 4/100) compared to the other platforms.

### Video quality assessment

**Fig. 3:**
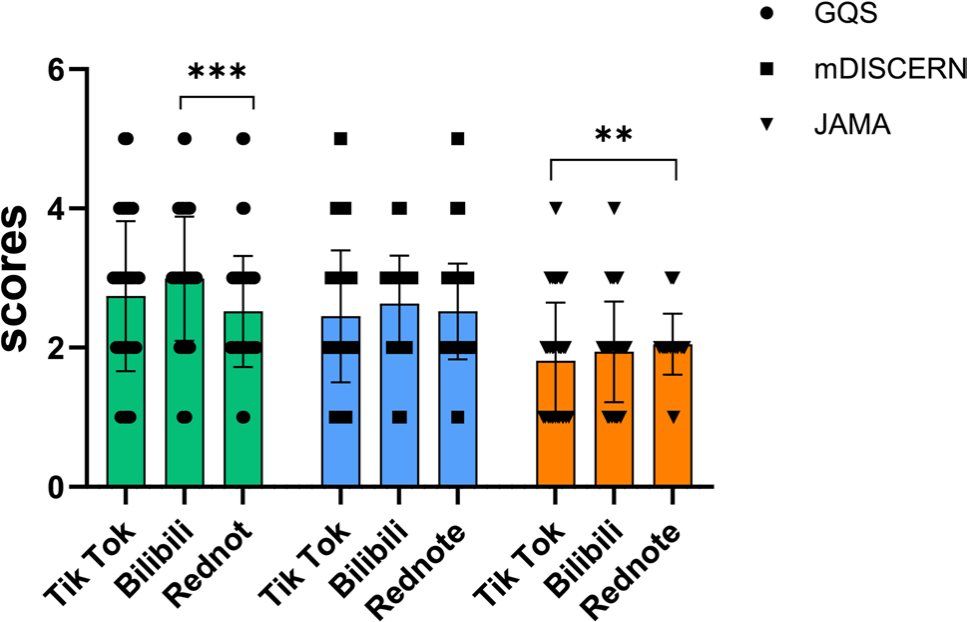
Comparison of GQS, mDISCERN, and JAMA scores across platforms. Differences among platforms were assessed using the Kruskal–Wallis test, followed by Dunn’s test for adjusted pairwise comparisons; *** indicates *p* < 0.001,** indicates *p* < 0.01

The Kruskal-Wallis test was used to compare the differences in the video quality scores among the three platforms. The results (Fig. 3) showed that GQS scores (*p* < 0.001, η² = 0.047) and JAMA scores (*p* = 0.012, η² = 0.024) differed significantly across platforms, with both effect sizes being small. In contrast, no statistically significant difference was found for mDISCERN scores (*p* = 0.131, η² = 0.007).Post-hoc pairwise comparisons revealed that for GQS scores, Bilibili had a significantly higher median score of 3 (IQR: 2–4) than Rednote’s median of 2 (IQR: 2–3) (adjusted *p* < 0.001). For JAMA scores, although both TikTok and Rednote had a median score of 2, their distributions differed. The post-hoc test indicated that Rednote’s JAMA scores were overall higher than those of TikTok (adjusted *p* < 0.01). Together with its narrower IQR (2–2) and higher mean score, this suggests that Rednote delivered more consistently higher quality on the JAMA.

**Fig. 4:**
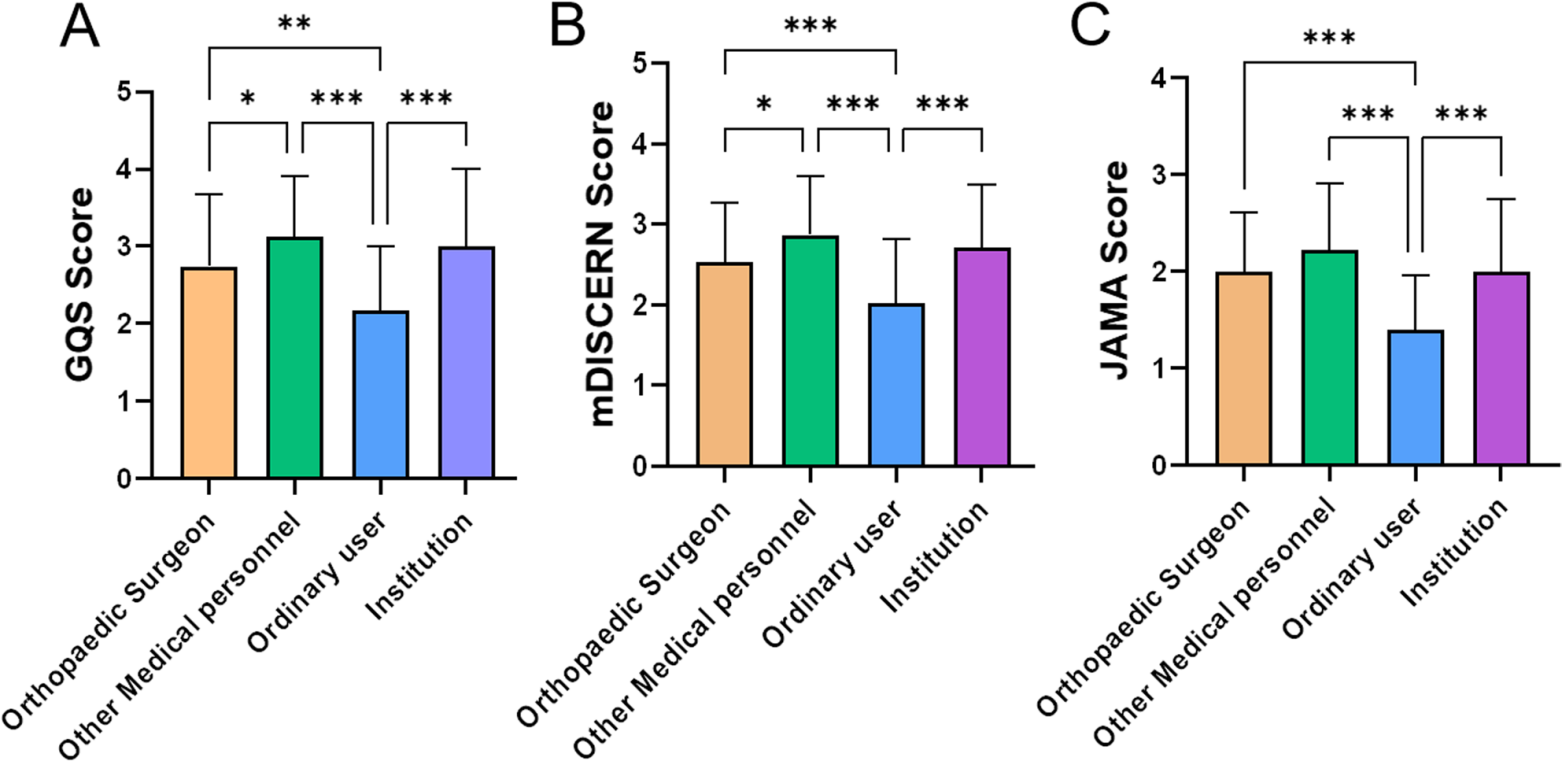
**A** Comparison of GQS scores among different publisher identities; **B** Comparison of mDISCERN scores among different publisher identities; **C** Comparison of JAMA scores among different publisher identities; the Kruskal–Wallis test followed by Dunn’s post-hoc test (adjusted *p*-values) was used for comparisons; *** indicates *p* < 0.001,** indicates *p* < 0.01 and * indicates *p* < 0.05

In addition, we analyzed and compared the differences in quality scores among the 300 short videos based on their uploader sources. As shown in Fig. 4, significant differences were observed in GQS scores (*p* < 0.001, η² = 0.098), mDISCERN scores (*p* < 0.001, η² = 0.099), and JAMA scores (*p* < 0.001, η² = 0.143), with effect sizes ranging from moderate to large.Post-hoc pairwise comparisons revealed that for the GQS score, the GQS score of ordinary users was significantly lower than that of Orthopaedic Surgeon (adjusted *p* < 0.01), other medical personnel (adjusted *p* < 0.001), and institutions (adjusted *p* < 0.001), while the GQS score of Orthopaedic Surgeon was also lower than that of other medical personnel (adjusted *p* < 0.05); for the mDISCERN score, the mDISCERN score of ordinary users was significantly lower than that of Orthopaedic Surgeon (adjusted *p* < 0.001), other medical personnel (adjusted *p* < 0.001), and institutions (adjusted *p* < 0.001), while the mDISCERN score of Orthopaedic Surgeon was also lower than that of other medical personnel (adjusted *p* < 0.05); for the JAMA score, the scores of ordinary users for publishing short videos were also significantly lower than those of Orthopaedic Surgeon (adjusted *p* < 0.001), other medical personnel (adjusted *p* < 0.001), and institutions (adjusted *p* < 0.001).

### Correlation analysis

**Fig. 5:**
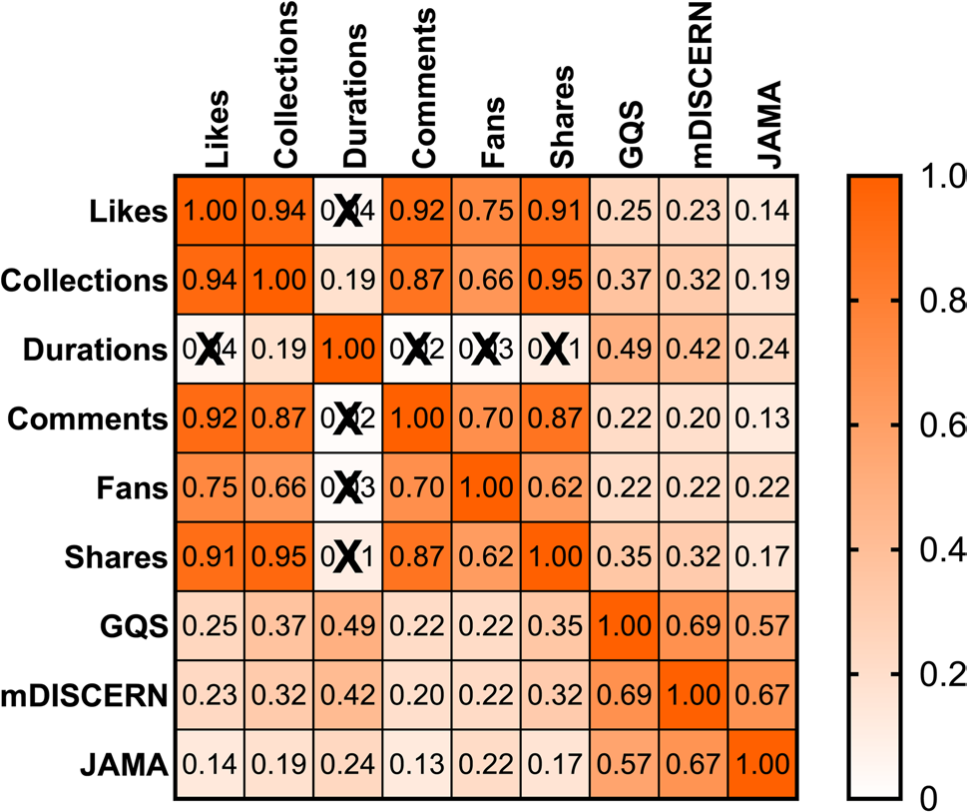
Correlation analysis between basic video characteristics and quality scores. |r|≤ 0.2 indicates no correlation; 0.2 <|r|≤ 0.4 indicates weak correlation;0.4 <|r|≤ 0.6 indicates moderate correlation;0.6 <|r|≤ 0.8 indicates strong correlation;|r|> 0.8 indicates very strong correlation;“X” represents *p* ≥ 0.05;all other values correspond to *p* < 0.05

To explore the relationship between the quality of short videos on knee osteoarthritis and their dissemination characteristics, this study employed the Spearman rank correlation analysis method. The detailed results are presented in Fig. 5. The main findings of the analysis are as follows: Firstly, the interaction indicators of the videos (likes, collections, comments, shares, and followers) show a very strong internal correlation, indicating that users’ online interaction behaviors are highly consistent. Secondly, the three quality assessment scales, GQS, mDISCERN, and JAMA, all present significant moderate to strong positive correlations with each other, further verifying that different assessment tools have good consistency reliability in this research context. The most core finding of the correlation analysis in this study is that there is only a weak or insignificant correlation between the information quality of the videos and their popularity (likes, collections, followers, etc.). Specifically, the correlations between all quality scores (GQS, mDISCERN, JAMA) and popularity indicators such as likes, comments, and followers are weak and mostly not significant. In contrast, there is a relatively clear positive correlation between video quality and video duration.

## Discussion

Short video platforms such as TikTok, Bilibili, and Rednote have become pivotal channels for public health information in the digital era.Leveraging their strong penetration, concise and vivid format, and interest-driven recommendation mechanisms powered by big-data algorithms, these platforms have effectively overcome the time and space limitations of traditional health education, demonstrating significant potential in raising public health awareness, disseminating basic medical knowledge, and promoting healthy behaviors [7, 30, 31].For chronic conditions such as KOA, which are highly prevalent, common, and involve diverse treatment approaches [32, 33], short videos—with their intuitive and accessible nature—serve as an efficient medium for demonstrating rehabilitation exercises, explaining disease progression, and sharing illness experiences. To a certain extent, they have filled the gap in traditional doctor-patient communication [34].

However, previous studies have found that the quality of short video information on social media vary widely in quality. Against this background, this study performed the first analysis of both the quality and basic characteristics of KOA-related content on mainstream short video multi-platforms in China, aiming to provide theoretical guidance for enhancing such content and assisting users in critically evaluating it.

### Main findings

The study revealed significant heterogeneity in the quality of KOA-related short videos across different platforms and among uploaders. A notable disconnect was observed between information quality and dissemination popularity. Furthermore, there remains considerable room for improvement in the overall quality of such content. These findings not only provide empirical evidence for understanding the patterns of health information dissemination on social media but also highlight pressing challenges and point to clear optimization pathways for public health practice.

### The basic characteristics of video dissemination

This study analyzed 300 videos from three major platforms: TikTok, Bilibili, and Rednote. The health information related to KOA short videos on these three platforms is abundant, including drug treatment, surgical treatment, case dissections, exercise therapy, patient case sharing, etc. These findings align with previous research on health information via social media, further confirming the considerable potential of short video platforms in health education [35–37].

The study founded that TikTok’s ecosystem is optimized for broad reach and high engagement, reflected in its significantly higher metrics for likes, shares, and creator followers. This aligns with its short-form, high-velocity content model. In contrast, Bilibili’s notably longer video format accommodates more systematic exposition, catering to its community’s preference for in-depth, knowledge-oriented content. This structural distinction underlies Bilibili’s relative advantage in comprehensive quality (GQS) scores, suggesting that platform architecture itself can enable or constrain informational depth.

Regarding the source of video publication, the study revealed an uneven distribution of uploader identities across platforms. On TikTok and Rednote, over 70% of videos were produced by medical professionals, likely reflecting stricter creator verification and growing public demand for credible sources.In contrast, Bilibili exhibited a more diverse creator ecosystem, with medical professionals accounting for only 40% of the content.Notably, the proportion of content published by medical professionals in this study (68% overall) is higher than that reported in previous studies on general health-related videos [38, 39]. This shift may indicate a trend toward content specialization in professional vertical fields with high barriers to entry, such as knee joint health, driven jointly by platforms and users. It also indirectly reflects the increasing public demand for professional and reliable health information.

### Quality assessment of the video

Previous studies have conducted quality information evaluations for diseases such as the spine, carpal tunnel syndrome, and diabetes [19, 35, 40, 41], but most of these studies focused on a single platform or only used a single video quality assessment tool.Notably, the prevalence of low-quality health videos raises important public health concerns, as such content may mislead viewers, promote inappropriate self-management, delay seeking professional care, or contribute to unnecessary anxiety, ultimately worsening disease outcomes and increasing health burdens. To enable a more comprehensive evaluation, this study employed three established tools—GQS, mDISCERN, and JAMA—to assess KOA-related videos across three major Chinese short-video platforms.

The overall quality scores of video information across all three platforms were suboptimal. Bilibili showed a significant advantage in the GQS score. The long-video format helps to present more systematic and comprehensive information.Notably, no significant difference was observed in mDISCERN scores across platforms, a tool emphasizing the fairness and clarity of uncertainty in treatment options [39, 42].The discrepancy between platform performance on different assessment tools highlights the risk of relying on a single metric for quality evaluation, as critical dimensions may be overlooked. These findings collectively indicate a common weakness in how short video platforms present health information, particularly in providing balanced treatment advice—an area requiring targeted improvement across all platforms.

By comparing the ratings of content from different publisher identities, it was found that the content posted by Orthopaedic Surgeon, other medical personnel, and institutions significantly outperformed that of ordinary users on all three quality scales. This is consistent with the conclusions of other health information studies [43].Interestingly, other medical professionals slightly surpassed Orthopaedic Surgeon on the GQS and mDISCERN scales. This may be attributed to the more comprehensive and practical guidance they provide in nonsurgical management—such as exercise therapy and lifestyle interventions—offering patients clearer and more actionable advice, thereby achieving higher ratings in terms of information quality and practicality.This highlights the importance of teamwork and comprehensive health education in KOA health promotion.

In conclusion, the research found that the three platforms have distinct characteristics. Bilibili focuses on the depth of content information, Rednote platform emphasizes the reliability of information, while TikTok pays attention to the breadth of information dissemination.

### The correlation between the basic characteristics of short videos and their ratings

Correlation analysis revealed only a weak or non-significant association between the information quality of short videos and their popularity, consistent with prior studies on conditions such as laryngeal cancer [44], gallstones [45], and liver cancer [46]. This suggests that popularity metrics, such as likes and shares, do not reliably reflect the informational quality of health-related content. This discrepancy may be attributed to the recommendation algorithms of short-video platforms, which often prioritize content that elicits emotional engagement or presents definitive conclusions, thereby amplifying its reach. Furthermore, general audiences may lack the expertise to evaluate information accuracy, making emotional appeal, presentation style, and creator relatability more influential factors in their engagement decisions.

At the same time, the positive correlation trend between video duration and quality in the study indicates that sufficient duration is a necessary condition for presenting complex health knowledge and conducting rigorous discussions. However, this puts such videos at a distinct disadvantage within the short-duration and entertainment-oriented paradigm of short video platforms, thus leading most videos with comprehensive content and rigorous logic to fail in effectively serving the purpose of health science popularization. In the future, social media platforms should refine their recommendation algorithms to better identify and promote high-quality health content, thereby enhancing their role in responsible health communication.

### Targeted public health recommendations

To optimize the KOA health information ecosystem and reduce the potential disease burden caused by misinformation, we propose a multi-level intervention framework encompassing platforms, content creators, health authorities, and the public.

At the platform level, algorithms need to be optimized to link information science quality (certification qualifications, authoritative citations, disclosure of interests) with interaction data, and to favor high-quality scientific content in terms of traffic; strengthen the qualification review and identification of creators, and implement standardized labels such as individual difference prompts for treatment advice videos; implement hierarchical management of content, raise the release threshold for high-risk content such as treatment recommendations, and set up expert review and reporting channels.

At the creator level, multidisciplinary collaboration in content production should be encouraged. Creators must transparently disclose their professional qualifications, any relevant conflicts of interest, and the date of publication. Treatment options should be presented objectively, outlining indications, potential benefits, and risks, while avoiding absolute claims or commercially biased promotion.

Health authorities and government agencies can lead by developing short-video communication guidelines and templates for chronic diseases like KOA, offering practical reference materials for creators. They could also use engaging video formats to improve public health literacy—teaching viewers to prioritize content from verified professionals, trace original sources, and remain cautious of oversimplified or definitive claims.

Through these coordinated efforts, the public can become more discerning consumers of health information. By recognizing markers of credibility and understanding the importance of source transparency, users will be better equipped to identify reliable content and reduce the impact of misinformation.

### Limitations

This study has certain limitations. Firstly, the sample was solely derived from three platforms and focused on KOA. The conclusions should be used with caution when extending to other platforms or disease fields. It should be noted that although a neutral account was used for the search, the default sorting algorithm within each platform may introduce biases related to popularity, freshness, or regional preferences. These potential biases could have affected the representativeness of the sample and thus constitute a source of algorithm-related bias. Secondly, although we used internationally recognized scales and reported good inter-rater consistency, the quality assessment inevitably includes subjective elements. Although the deviation can be controlled to some extent through the examination of multiple raters, it cannot be completely eliminated. Thirdly, this study focused on content quality and dissemination characteristics but did not assess the actual impact of videos on user behavior, such as whether users adopt recommended treatments or seek professional advice, which represents a gap requiring further investigation. Finally, this study mainly focused on the content dimension of the information and failed to deeply analyze the impact of non-textual factors such as the narrative style, visual presentation, and musical emotion of the videos on user trust and behavioral intentions. This is an important direction for future research.

## Conclusion

This study systematically evaluated the quality and reliability of KOA-related short videos across three mainstream Chinese platforms, revealing significant heterogeneity in quality across platforms and uploaders, a disconnect between quality and popularity, and room for overall improvement. The key contributions of this study are threefold: (1) Providing the first multi-platform, multi-tool assessment of KOA short-video quality in China; (2) Identifying platform-specific characteristics (e.g., TikTok’s high engagement, Bilibili’s in-depth content, Rednote’s transparent information) and uploader-related quality differences; (3) Highlighting the public health challenge of popularity not equaling quality in health information dissemination. Clinically, these findings emphasize the need for medical professionals to play a leading role in short-video health education, especially in providing balanced treatment information. From a public health perspective, the multi-party collaborative framework—platform algorithm optimization, creator standardization, public health literacy improvement—proposed in this study can help transform short-video traffic advantages into effective KOA prevention and control efforts. Future research should focus on user behavior impact and content optimization strategies tailored to different platforms.

## Supplementary Information

Supplementary Material 1.

Supplementary Material 2.

Supplementary Material 3.

Supplementary Material 4.

## Data Availability

The raw video metadata (e.g., likes, comments, duration) and quality assessment scores (GQS, mDISCERN, JAMA) generated during the study are not publicly available due to platform content copyright and user privacy restrictions, but are available from the corresponding author upon reasonable request.
