# Supplementary material for: Quality and reliability of knee osteoarthritis-related information on short video platforms in China: a multi-method cross-sectional study
Source: BMC Public Health. 2026 Feb 2;26:770. doi: 10.1186/s12889-026-26455-9 (PMC12955160; doi:10.1186/s12889-026-26455-9)
Supplement: Supplementary file 1 — Supplementary Material 1. [file 12889_2026_26455_MOESM1_ESM.pdf]

Supplementary Table 1. Global Quality Score (GQS) (Scoring ranges from 1 to 5)

GQS Definition	Score
Poor quality, poor flow of the video, most information missing, not at all useful for patients	1
Generally poor quality and poor flow, some information listed but many important topics missing, of very limited use to patients	2
Moderate quality, some important information is adequately discussed	3
Good quality good flow, most relevant information is covered, useful for patients	4
Excellent quality and flow, very useful for patients	5
