# Supplementary material for: Quality and reliability of knee osteoarthritis-related information on short video platforms in China: a multi-method cross-sectional study
Source: BMC Public Health. 2026 Feb 2;26:770. doi: 10.1186/s12889-026-26455-9 (PMC12955160; doi:10.1186/s12889-026-26455-9)
Supplement: Supplementary file 2 — Supplementary Material 2. [file 12889_2026_26455_MOESM2_ESM.pdf]

Supplementary Table 2. Modified DISCERN benchmark criteria.

Score*	Reliability Score
1 score	The video was clear, concise and understandable
1 score	The information sources were reliable
1 score	The information presented was balanced and unbiased
1 score	Additional sources of information were provided for patient reference
1 score	Areas of uncertainty or controversy were appropriately addressed.

*The criteria of each aspect were scored separately, and 1 point was accumulated when the criteria were reached. A total reliability score ranging from 0 to 5 was obtained.
