# Supplementary material for: Quality and reliability of knee osteoarthritis-related information on short video platforms in China: a multi-method cross-sectional study
Source: BMC Public Health. 2026 Feb 2;26:770. doi: 10.1186/s12889-026-26455-9 (PMC12955160; doi:10.1186/s12889-026-26455-9)
Supplement: Supplementary file 3 — Supplementary Material 3. [file 12889_2026_26455_MOESM3_ESM.pdf]

Supplementary Table 3. The Journal of the American Medical Association (JAMA) benchmark criteria.

Score*	Score component	
1 score	Authorship	Provided authorship information
1 score	Attribution	Listed copyright information and references/sources
1 score	Currency	Included the initial date and subsequent updates
1 score	Disclosure	Disclosed any potential conflicts of interest, funding, sponsorship, advertising support or video ownership

*The criteria of each aspect were scored separately, and 1 point was accumulated when the criteria were reached. A total reliability score ranging from 0 to 4 was obtained.
