# Supplementary material for: Quality and reliability of knee osteoarthritis-related information on short video platforms in China: a multi-method cross-sectional study
Source: BMC Public Health. 2026 Feb 2;26:770. doi: 10.1186/s12889-026-26455-9 (PMC12955160; doi:10.1186/s12889-026-26455-9)
Supplement: Supplementary file 4 — Supplementary Material 4. [file 12889_2026_26455_MOESM4_ESM.pdf]

# Supplementary Material: Content Classification Criteria and Inter-Rater Reliability Analysis

**1. Content Classification Criteria**

Content classification was defined as follows to ensure clear boundaries and reproducibility:

(1) Disease education and assessment: Coverage of the etiology, clinical symptoms, diagnostic criteria, risk factors, and disease staging of knee osteoarthritis (KOA);

(2) Surgical treatment: Information related to surgical indications, operative procedures, postoperative care measures, and clinical efficacy of KOA surgery;

(3) Drug therapy: Introduction to types of KOA-related drugs, dosage and administration methods, potential side effects, and key precautions for use;

(4) Anatomical pathology: Explanations of knee joint anatomical structure, pathological changes associated with KOA progression, and underlying pathogenic mechanisms;

(5) Patient case sharing: Personal narratives of KOA diagnosis experiences, treatment processes, and rehabilitation journeys;

(6) News advertisements: Commercial promotions for KOA-related treatments (e.g., drugs, medical devices, health products) and news reports relevant to KOA.

Classification was performed independently by two researchers (X.D. and T.M.). In cases of discrepancies in classification results, a third senior researcher (Y.W.) reviewed the disputed content, and a final consensus was reached through group discussion to ensure the accuracy of classification.

**1. Assessment Process**

To ensure objectivity, two evaluators (Rater T.M, and Rater Y.W.) independently rated the quality of all included videos. Before assessment, both raters received unified training on the GQS, mDISCERN, and JAMA tools, and participated in calibration discussions to align their understanding of the scoring criteria. If the two raters disagreed on the rating for a given video, a third senior researcher reviewed the case, and a final consensus score was reached through discussion among the three.

**2. Reliability Analysis Methods**

To quantify the consistency between the two primary raters (Rater T.M, and Rater Y.W.), appropriate statistical measures were selected based on the data type of each scale:

GQS scores, being ordinal categorical variables, were analyzed using the Weighted Cohen's Kappa coefficient.mDISCERN and JAMA scores were treated as continuous variables in the analysis. Their reliability was assessed using the Intraclass Correlation Coefficient (ICC).

**3. Reliability Results**

The two raters demonstrated excellent agreement across all three tools, with the following reliability coefficients:

GQS: Weighted Kappa = 0.832 (95% CI: 0.78 – 0.88)

mDISCERN: ICC = 0.802 (95% CI: 0.75 – 0.85)
JAMA: ICC = 0.922 (95% CI: 0.90 – 0.94)

**4. Interpretation**

According to widely accepted benchmarks:

A Kappa value above 0.81 for GQS indicates almost perfect agreement between raters.

ICC values above 0.75 for both mDISCERN and JAMA reflect good to excellent inter-rater reliability.

These results confirm that the assessment procedure and tools used in this study were highly reliable, and that the independent ratings provided by the two primary evaluators were consistent and credible, establishing a solid methodological foundation for subsequent data analysis.
